# Liver, Oxidative Stress and Metabolic Syndromes

**DOI:** 10.3390/nu13020301

**Published:** 2021-01-21

**Authors:** Rita Rezzani, Caterina Franco

**Affiliations:** 1 Anatomy and Physiopathology Division, Department of Clinical and Experimental Sciences, University of Brescia, 25123 Brescia, Italy; c.franco@studenti.unibs.it; 2 Interdipartimental University Center of Research “Adaption and Regeneration of Tissues and Organs-(ARTO)”, University of Brescia, 25123 Brescia, Italy

Today, talking about metabolic syndrome (MetS) and oxidative stress, can be risky. This is not because there is little to say or a lack of information, but because scientists can end up being repetitive and falling into stereotypes.

Many studies and works have focused on this increasingly emerging issue, in particular, considering the fact that many of the conditions that define MetS, especially obesity, are defined as part of a sort of worldwide epidemic. In any case, what is of greater interest is the clinical applicability the latest knowledge [1,2].

Since 2001—the year in which MetS was clearly defined for the first time—the situation has worsened: MetS and the associated risk factors now represent a major public health concern. They may be considered a social problem and also as the common basis for a large number of chronic illnesses that are exacerbated and triggered by excessive body weight, dyslipidemia, hypertension, type 2 diabetes, and atherosclerosis [1].

Recent studies, however, have demonstrated a progressive approach: they have focused not just on the individual, isolated diseases, but also on the underlying metabolic imbalance.

They have also investigated the molecular and cellular mechanisms considered to be the main determinants of the imbalance itself. This has enabled the association of oxidative stress as one of the factors causing MetS. Next to this, they have also identified a number of soluble factors linked with sedentary life, abdominal obesity and different metabolic pathways that could represent a target point for novel preventive and treatment strategies [1].

Moreover, recently research has also gone beyond the most well-known pathologies closely associated with metabolic imbalance [3], focusing on the impact that this imbalance could have on each of the organs.

Starting with the basics, metabolism can be defined as a group of biochemical reactions that transform nutrients into the energy necessary for life. Similarly, MetS could be briefly summarized as an imbalance in this metabolism. This means that obesity is not the only feature ascribable to MetS; on the contrary, this irregularity is manifested also in other pathological forms. Moreover, the main manifestations of the MetS, from abdominal obesity, dyslipidemia to insulin resistance and hypertension are still considered as major risk factors for the development of diabetes mellitus and systemic cardiovascular diseases (CVDs) [1,3].

Low-grade, chronic inflammation, associated with a low-grade, whole-body increase in oxidative stress, is the basic weakness in human health; and this inflammatory state origins mainly from the rise in reactive oxygen species (ROS).

The loss of metabolic balance has an impact in many ways and have also many side effects.

Obesity, in this case, is identified with the expansion of the abdominal fat mass, in particular in the visceral area, leading to what is known as “adiposopathy”. This is, specifically, one of the central points in the development of CVDs. Next to this, obesity is also associated with an abnormal production of adipokines that are the main causes of the characteristic insulin resistance and lipid disorders [1].

For this reason, mainly considering MetS effects on glucose pathways, this syndrome has often been analyzed from an endocrinological point of view: the International Diabetes Foundation (IDF) gave a definition of MetS as the combination of clinical and metabolic factors, including insulin resistance, hyperglycemia, hypertension, dyslipidemia, and abdominal obesity, paying particular attention to abdominal obesity [2].

In addition, more recent hypothesis tried to explain the metabolic disturbances focusing on the secretive and productive loss of balance. In particular, more emphasis has been placed on myokines or “exercise factors” such as interleukin (IL)-6, IL-15, and irisin that are strongly upregulated during aerobic physical exercise. They are extremely important, in particular in the management of the adipocytes: irisin, for example, induces “browning” of white adipose tissue and results in increased energy expenditure and body weight reduction [1].

In the end, also β-aminoisobutyric acid (BAIBA), a non-protein amino acid secreted by skeletal muscles has been defined as an endogenous protective myokine thanks to its capability to regulate adipose tissue browning and improve insulin sensitivity. This last point could be of central importance, in particular focusing on the biological effects that could be useful in the prevention and treatment of MetS [1].

The pathogenesis underlying MetS is very complex and is not yet clearly defined. In any case, the data collected up to now, reveal that oxidative stress plays a fundamental role in the evolution and progression of MetS. The results are not so clear, but an increase in biomarkers of oxidative stress and a decrease in antioxidant defense have been found in the blood of patients with MetS, suggesting that oxidant and antioxidant imbalance may play an important role in its manifestation.

In a physiological condition, equilibrium between the generation of ROS and reactive nitrogen species (RNS) and antioxidant levels enables cellular crosstalk and also the control of intracellular functions, as well as cell to cell interactions, proliferation, differentiation, migration, and contraction. Additionally, all these processes are normally carried out thanks to the direct or indirect reversible-redox modification of critical targets [4].

The problem starts with the pathological condition. During inflammation, atherosclerosis, ischemia and reperfusion injury, or diabetes mellitus, the oxidant-generating enzymes in tissues overwork, producing higher amounts of ROS and RNS, accumulating them in non-physiological locations. This additional productivity, associated with the lack of response from the antioxidant systems, leads to the irreversible oxidation of proteins, lipids, and nucleic acids; and this is what causes the oxidative stress [4].

But what is more important is that many studies, both in humans and in animals, have found that most MetS-related diseases cause mitochondrial oxidative stress, altered mitochondrial morphology and oxidative phosphorylation functions, directly and indirectly inducing mitophagy and apoptosis [5,6]. Moreover, the real importance of that discovered up to now is the possibility of translating it into defined in a therapeutic strategy. Reduced levels of reduced glutathione (GSH) have been measured in the blood plasma as well as in the red blood cells (RBCs) of MetS patients, as well as the reduced glutathione and glutathione disulfide (GSH and GSSG, respectively) in ratio animal models of obesity. The new interventions aim to reduce oxidative stress by acting on the antioxidant defense to restore redox balance and consequently to reduce cardiac dysfunction in MetS and related CDVs [4].

Oxidative stress is considered one of the causing factors of liver damage [6]. Many etiological aspects have been associated with excessive ROS production and the forthcoming liver diseases. Even if the pathogenesis of MetS is not so clear and well understood, studies have demonstrated that a high-fat-diet and an excessive nutrient intake could result not only in adipose tissue triglyceride accumulation with adipocyte typical hypertrophy and pro-inflammatory cytokines release, but also in ectopic fat deposition [7].

Specifically, an excessive lipid deposit in the liver can lead to steatosis, starting the road of no return to the non-alcoholic fatty liver disease (NAFLD) which is considered the hepatic manifestation of MetS [8].

Among all the hepatic diseases, NAFLD is probably the most prevalent chronic liver disorder [2,3,9]. Results show that, in this context MetS and specifically the resulting insulin resistance (IR), play an important role in the pathogenesis of NAFLD in obese patients. High glucose concentrations promote hepatic triglyceride synthesis, contributing to triglyceride accumulation.

For this reason, a better definition of MetS and of how it works could also be useful in preventing the onset of NAFLD [8,9].

Liver is also responsible for iron balance and this pathway is often compromised in MetS-related disease. In particular, studies underlined that the iron-induced liver injury could be explained by the increase of ROS level, finding in this way, also a correlation between liver disease and MetS pathogenesis [10].

Intervening in the management of MetS effects on the liver is crucial, particularly considering that liver diseases have an important impact on the homeostasis of the entire organism and that chronic liver disease represents an underestimated world public health problem [4,11].

Recent references confirm the relevance of this subject, counting, to date, about 57,000 articles in response to the search “metabolic syndrome” on PubMed.

What has continued to push the boundaries of research has been the will and the need to find the mechanisms underlying complex syndromes such as MetS.

Understanding how things work at cellular level, in fact, allows us to define a specific and personalized path of intervention and therapy. It also allows us to understand how the pathology could be linked—or could be itself the cause—of other systemic pathologies [12]

MetS and oxidative stress can be considered to be two separate problems, but it is even more difficult to deal with them considering their long-term consequences.

Atherosclerosis, for example, according to recent studies, is one of the leading causes of mortality and morbidity worldwide [13]. A recent update from the American Heart Association (AHA) in 2015 states that approximately 17,300,000 deaths worldwide each year are due to CVDs [13].

In addition, mood and neurological disorders (e.g., bipolar disorder, major depression, and Parkinson disease) share with MetS the same low-grade inflammation, showing increased levels of pro-inflammatory cytokines and acute phase proteins, higher level of lipid peroxidation with clear augmentation, and oxidized low density lipoprotein cholesterol (LDL-c), all associated with low levels of antioxidants.

Overall, the aim of this special issue. Despite the progress made in understanding and knowledge, a lot remains to be done. Many are the fields that have to be reassessed in a new light and many are the links that today are still undisclosed, but that need to be highlighted.

## Abbreviations

MetS	Metabolic Syndrome
CVDs	Cardiovascular Diseases
ROS	Reactive Oxygen Species
IDF	International Diabetes Foundation
IL-	Interleukin-
BAIBA	β-Aminoisobutyric Acid
RNS	Reactive Nitrogen Species
RBCs	Red Blood Cells
GSH	Glutathione
GSSG	Glutathione Disulfide
AHA NAFLD	American Heart Association Non-alcoholic Fatty Liver Disease
IR	Insulin Resistance
LDL-c	Low Density Lipoprotein Cholesterol T Lineage Progenitor Cells
